# Development and validation of the 23-item preterm birth risk assessment scale-Korean version

**DOI:** 10.1186/s12884-023-05975-x

**Published:** 2023-09-16

**Authors:** Jeung-Im Kim

**Affiliations:** School of Nursing, College of Medicine, Women Health Nursing, Soonchunhyang University, 31 Soonchunhyang 6-Gil, Dongnam-Gu, Cheonan City, 31151 Chungnam Province Korea

**Keywords:** Factor analysis, Statistical, Premature birth, Psychometrics, Scale development

## Abstract

**Background:**

Preterm birth (PTB) is a complex and significant challenge in obstetrics. Thus, clinicians and researchers have paid a keen interest in the identification of women at a high risk for PTB. This study aimed to develop a PTB risk assessment scale based on the preliminary 32-item Preterm Birth Risk Assessment Scale-Korean version (PBRAS-K).

**Methods:**

We enrolled 298 participants (167 in the exploratory factor analysis group from March 3, 2021 to August 31, 2021 and 131 in the confirmatory factor analysis group from December 3, 2021 to February 14, 2022) who delivered before 37^+0^ weeks after experiencing preterm symptoms and were admitted to high-risk pregnancy maternal–fetal intensive care units (MFICUs). After an item-reduction process in the exploratory factor analysis, the psychometric property scales were assessed using SPSS Statistics version 27.0, and the confirmatory factor analysis was conducted using AMOS version 27.0.

**Results:**

The Kaiser–Meyer–Olkin (KMO) test and Bartlett’s χ^2^ test of sphericity confirmed the adequacy of the sample for factor analysis (KMO = .81 (> .80), χ^2^ = 1841.38, *p* < .001). The final version of the PBRAS-K comprised 23 items within seven dimensions. Factor analysis identified items explaining 65.9% of the total variance. The PBRAS-K achieved a mean score of 35.58 (± 10.35) and showed high internal consistency and satisfactory reliability (Cronbach’s alpha = .85). Regarding concurrent validity, the PBRAS-K exhibited a low-to-moderate correlation with the PTB risk (*r* = .45, *p* < .001). As for criterion validity and convergent validity, the PBRAS-K showed a positive and high correlation with the Somatic Awareness Scale with Spontaneous Preterm Labor (SPL-SAS) (*r* = .65, *p* < .001) and pregnancy-related stress (*r* = .57, *p* < .001), respectively. Risk scoring for preterm delivery and SPL-SAS were moderately correlated (*r* = .53, *p* < .001).

**Conclusions:**

PBRAS-23-K is a valid and reliable instrument for assessing the risk for PTB in pregnant women. Clinical nurses are encouraged to apply and obtain information regarding effective interventions in MFICUs. This scale provides meaningful results and reflects the opinions of women who had experienced PTB. The PBRAS-23-K should be evaluated for standardization and cut-off scores using larger sample sizes in the future.

**Supplementary Information:**

The online version contains supplementary material available at 10.1186/s12884-023-05975-x.

## Background

Preterm birth (PTB), defined as the birth of a baby at <37 weeks of gestation [1, 2], is the second most common cause of infant death in the United States [3]. Preterm infants may be born with serious health problems, some of which can be lifelong (e.g., cerebral palsy). Other problems such as learning disabilities may emerge later in childhood or adulthood. PTB is one of the most complex and significant challenges in obstetrics. Thus, clinicians and researchers have paid a keen interest in the identification of women at a high risk for PTB [4].

Several clinicians have attempted to predict the incidence of PTB; nonetheless, PTB remains difficult to predict. According to the American Association for Clinical Chemistry guidelines, biomarkers such as interleukin-6, placental alpha macroglobulin-1, and fetal fibronectin have low positive predictive values and provide limited utility in the diagnostic algorithms most commonly applied in the United States [3]. Approximately 50% of PTBs follow spontaneous preterm labor (PTL), whereas approximately 35% follow preterm premature rupture of the membranes; the remaining PTBs are iatrogenic and are due to medically induced maternal or fetal complications [4, 5]. Approximately 50% of PTBs occur after natural PTL. Thus, if such PTL can be prevented, this will greatly contribute to a reduction in PTB occurrence.

PTB prediction involves a screening test with high sensitivity and high negative predictive value. Various screening tools can be classified into four groups—namely, monitoring of uterine activity, assessment of cervical length, measurement of cervical fetal fibronectin, and detection of the presence of bacterial vaginosis in early pregnancy [5]. While these screening tools are useful in hospitals, an assessment scale that can be used for community-dwelling pregnant women has not yet been developed, and only a few measurements for PTB or PTL assessment are available. Creasy et al. developed a system for scoring the risk for preterm delivery (RPD) and predicting spontaneous PTB. The RPD assessment was divided into four components: socioeconomic status, medical history, daily habits, and aspects of the current pregnancy. The RPD system was better for multigravida women, and rescoring at 26–28 weeks of gestation increased the predictive accuracy [6]; however, its overall predictive value ranged only from only 17% to 34%. In contrast to RPD, the screening tool for the risk for PTB developed by Cho and Kim (2020) considered the biomedical and somato-psychological risks of pregnant women in Korea [7]. Thus, it comprised psychological questions that were not included in the RPD assessment. Kim [8] identified nine components, which included obstetrical and physical states, medical problems, life-related stress, pregnancy-related stress, spousal support, and information support. PTL is a biopsychosocial process that does not occur in isolation because of individual factors; rather, it is a combination of factors that increase the risk for PTB [9]. Psychological risk factors of PTL are broad and diverse and have been shown to influence the rate of premature birth [10, 11]. Cumulative stress increases the risk for PTL [12]. Family support is known to affect PTL rates and is generally considered a protective factor [13]. These findings suggest that biopsychosocial stressors should be included in the assessment of PTL risk.

Therefore, a scale that can inform clinicians and pre-pregnant women about the causes of PTB must be developed to obtain proper care. Based on the nine components of PTL and PTB [8], the author developed a preliminary 32-item Preterm Birth Risk Assessment Scale-Korean version (PBRAS-32-K) [14] in the first stage of the scale development process. The present study focused on the second stage of the scale development process (i.e., psychometric evaluation of the PBRAS-32-K) [14].

## Methods

### Study design

This methodological study adopted a cross-sectional survey design to evaluate the reliability and validity of the PBRAS-32-K [14] according to the guidelines for scale development [15].

### Sample

Offline data collection was planned for antepartum women who had experienced PTL at each maternal-fetal intensive care unit (MFICU) center; however, this was changed to an online Google platform because of the coronavirus disease pandemic. One hundred forty participants completed a questionnaire at the outpatient clinic of the hospital, and others completed it on an online platform. With the cooperation of the heads of seven MFICUs and head nurses, a recruitment notice was posted on the bulletin board. Pregnant women responded to the questionnaire using QR codes. The participants provided informed consent for their participation on the platform, verifying that they understood the purpose and content of the study. The required sample size was over 300, or five to ten times the number of exploratory factor analysis (EFA) items and five times the number of final confirmatory factor analysis (CFA) items [16]. The researcher adopted five times the number of items for both EFA and CFA in this study. Therefore, a minimum of 160 and 115 participants were required for EFA and CFA, respectively. Data collection was completed upon obtaining a sufficient number of samples. After excluding three sets of insufficient data on the PBRAS-32-K, a total of 298 responses (167 for 32-item EFA and 131 for CFA) were used. At least five participants were required for each item of the scale to ensure construct validity, and data on a representative sample of the target population were collected to ensure construct validity and reliability [15]. The inclusion criteria were as follows: (i) pregnancy before 37 weeks of gestation, (ii) PTL, and (iii) consent to participate. Postpartum women were excluded from the study. The exclusion criteria were as follows: (i) pregnancy after 37^+0^ weeks, and (ii) pregnancy with no symptomatic problems. The offline survey was administered in an outpatient setting. It was limited to outpatient surveys because there was a concern that answering the questionnaire could be stressful for hospitalized women and that this could lead to increased preterm labor. The offline survey was conducted at hospitals with MFICUs across the country.

### Data analysis

**Fig. 1:**
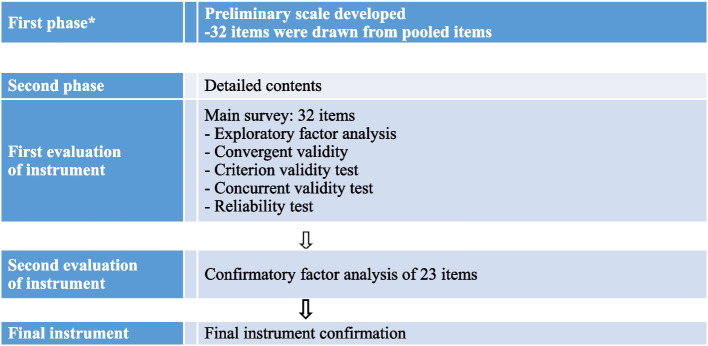
Evaluation of the PBRAS-32-K. *[14]

PBRAS-32-K was evaluated with respect to factors, reliability, and validity. The second stage of PBRAS-32-K development, except for the first stage, is shown in Fig. 1. Reliability and validity tests for the descriptive statistics and psychometrics were performed using IBM^®^ SPSS^®^ Statistics 27^TM^, and IBM^®^ SPSS^®^ 27^TM^ (Chicago, IL) was used for model fitting. Descriptive statistics were used to determine the frequency, range, mean, and standard deviation of the participants’ demographic and clinical characteristics. All other tests were two-tailed, and a *p*-value of <5% was considered to be statistically significant. Item analysis included mean and standard deviation, skewness and kurtosis, and corrected item-total correlation coefficient. The absolute skewness and kurtosis values were evaluated to determine whether they were less than 3.0 for skewness and less than 7.0 for kurtosis, satisfying the item analysis conditions. Data were automatically analyzed using SPSS version 27.0. Additionally, the total scores and intra-item correlations were analyzed to determine whether the values were ≥.30 (a correlation coefficient of ≥.30 was adequate if the same concept was measured by several items) [17, 18].

### Assessment instrument

#### Construct validity testing

EFA and CFA were conducted to assess construct validity. Principal component factor analysis was performed as a factor extraction model to minimize information loss from the minimum-factor prediction, and varimax rotation was run to clearly classify the factors by maximizing the sum of the factor-loading variance. First, Kaiser-Meyer-Olkin (KMO) test and Bartlett’s test of sphericity were used to confirm the suitability of materials for EFA [19]. The KMO measure of sampling adequacy was ≥.5, indicating that the sample selection was adequate for factor analysis. Bartlett’s test of sphericity confirmed the patterned relationships between the variables, as seen in the correlation matrix (*p* <.001). Bartlett’s test of sphericity tested the hypothesis that the correlation matrix was an identity matrix, which would indicate that the variables were unrelated, and therefore, unsuitable for structure detection. Small significance level values (i.e., <.05) indicated that factor analysis of the data might be useful [17].

For the extraction of factors via the EFA, the number of factors was determined using the following criteria: eigenvalue of ≥1, factor loading of ≥.40 [19], and accumulative variance of 50%–60.0% [20]. For the verification of the CFA model, the following were verified: goodness-of-fit coefficients (≥.9), normed χ^2^ (χ^2^/df), normed fit index (NFI ≥.9), relative fit index (RFI ≥.9), incremental fit index (IFI ≥.9), goodness-of-fit index (≥.9), standardized root mean residual (≤.05), root mean square error of approximation (RMSEA ≤.05), Tucker-Lewis index (TLI ≥.9), and comparative fit index (CFI ≥.9) [17, 20].

#### Reliability evaluation

The reliability of the scale was assessed and the final 23 items were selected based on the measurement of internal consistency using Cronbach’s alpha.

#### Validity evaluation

1. Convergent validity

For convergent construct validity [21], the PBRAS-23-K was compared with pregnancy-related stress using Pearson’s correlation coefficient because PTB screening had been previously reported to show a moderate positive correlation with pregnancy-related stress [7] and PTB was related to stress [12, 22].

2) Concurrent validity

For concurrent validity, the PBRAS was compared with the RPD [6] scoring system, which is the gold standard used in obstetric studies, because the PBRAS measures different constructs of the RPD (the RPD has four constructs—namely, “socioeconomic status,” “previous medical history,” “daily habits,” and “aspects of the current pregnancy”); however, the Cronbach’s alpha has not yet been reported.

3) Criterion validity

Finally, the criterion validity of the PBRAS was evaluated using the Somatic Awareness Scale with Spontaneous Preterm Labor (SPL-SAS) [23]. The SPL-SAS has the constructs of “physical tension sensations,” “traditional PTL sensations,” “psychosomatic sensations,” “sickness,” “vaginal discharge,” “gastrointestinal sensations,” “gastrointestinal irritability,” and “energy sensation,” with a Cronbach’s alpha of .94 [23]. Correlations were high at *r* > .6 [24].

### Ethical considerations

Ethical clearance was obtained annually from the Institutional Review Board (IRB) of Soonchunhyang University (reference numbers 1040875-201905-SB-026, 202001-SB-004-05 [second year] and 202101-SB-009-01 [third year]). All participants provided informed consent to participate in the study. Documents for agreement regarding online and offline surveys were submitted to the IRB office and approved. After data coding, all personal data were deleted.

## Results

The general characteristics and details of the participants are summarized in Table 1. A total of 298 women who delivered before 37^+0^ weeks after experiencing preterm symptoms and were admitted to an intensive care unit for high-risk pregnancy were included in this study. Data from 298 participants were analyzed, excluding the insufficient data from one participant. Regarding sociodemographic characteristics, participants in the EFA group had a mean age of 34.4 (±4.50) years, height of 160.7 (±5.53) cm, and pre-pregnancy weight of 57.76 (±11.32) kg, and the mean age of their husbands was 37.2 (±5.03) years. On the other hand, participants in the CFA group had a mean age of 34.7 (±4.32) years, height of 161.5 (±5.16) cm, and pre-pregnancy weight of 60.59 (±11.53) kg, and the mean age of their husbands was 37.1 (±4.77) years. Overall, 75 (45.5%) and 27 (16.2%) participants in the EFA group and 45 (34.4%) and 12 (9.2%) participants in the CFA group had experience with PTB and preterm premature rupture of the membranes, respectively. Furthermore, PTB was expected in 31 (18.6%) participants in the EFA group and 31 (23.8%) participants in the CFA group (Table 1).

**Table 1:** Sociodemographic and obstetric characteristics (*N* = 298)

Characteristics	Categories	First survey (*n* = 167)	Second survey (*n* = 131)
		**M ± SD or *n* (%)**	**M ± SD or *n* (%)**
Women’s age (years)		34.4 ± 4.50	34.7 ± 4.32
Husbands’ age (years)		37.2 ± 5.03	37.1 ± 4.77
Education	Middle junior	2 (1.1)	1 (0.7)
	High school	24 (14.4)	38 (29.0)
	University	112 (67.1)	82 (62.7)
	Graduate school	29 (17.4)	10 (7.6)
Women’s height (cm)		160.70 ± 5.53	161.5 ± 5.16
Women’s pre-pregnancy weight (kg)		57.76 ± 11.32	60.59 ± 11.53
Previous preterm birth (PTB)	No	90 (54.5)	86 (65.6)
	Yes	75 (45.5)	45 (34.4)
Preterm premature rupture of the membranes before PTB	No	140 (83.8)	119 (90.8)
	Yes	27 (16.2)	12 (9.2)
Participants’ expectation for PTB	No	136 (81.4)	100 (76.2)
	Yes	31 (18.6)	31 (23.8)
Absolute bed rest meaning by explanation	Know	136 (81.4)	116 (88.5)
	Did not know	31 (18.6)	15 (11.5)
Adequate information from the health team	Dissatisfied	31 (18.6)	26 (19.8)
	Satisfied	136 (81.4)	105 (80.2)
Compliance with instruction	No	154 (92.2)	122 (83.1)
	Yes	13 (7.8)	9 (6.9)

### Construct validity

Psychometric analysis was performed using the EFA, CFA, concurrent validity, criterion validity, internal consistency, and item-total correlation.

### Sampling adequacy by the KMO test and Bartlett’s test of sphericity

**Fig. 2:**
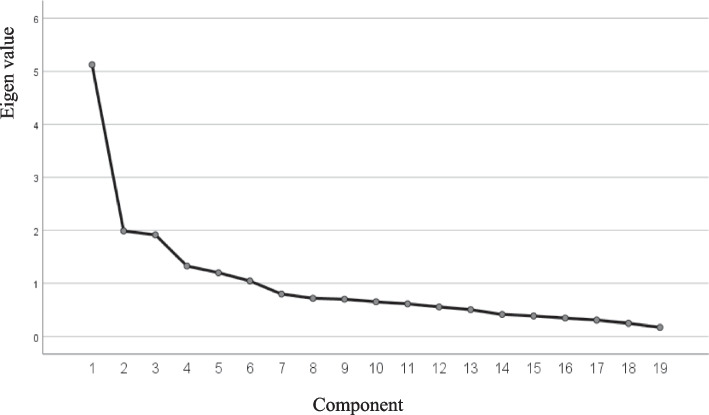
Scree plot

To assess construct validity, the researcher conducted an EFA with 32 items on 167 pregnant women. First, sampling adequacy was evaluated using the KMO test and Bartlett’s χ^2^ test of sphericity, which confirmed that this sample was adequate for factor analysis [25] (KMO = .81 (>.80), χ^2^ = 1841.38, *p* < .001). The number of factors was determined using a scree plot (Fig. 2) and the minimum average partial because the Kaiser rule tends to severely overestimate the number of factors. The EFA revealed seven factors.

Absolute skewness and kurtosis values by SPSS version 27.0 were normally distributed. All absolute values were less than 3.0 for skewness (0.2–1.41) and less than 7.0 for kurtosis (0.10–1.62), satisfying the item analysis conditions. In the pattern matrix, the average factor loading distributed from 0.63 to 0.77 and was higher than 0.70 (Table 2).

**Table 2:**
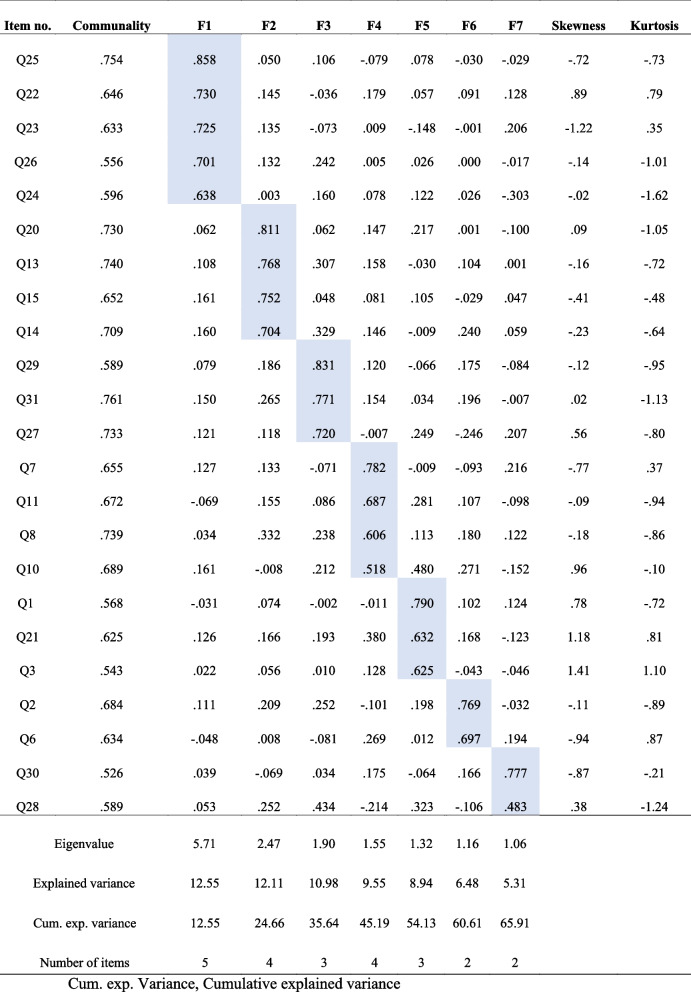
Fifth factor analysis of PBRAS-K (seven factors, 23 items) *(N* = *167)*

### Item communality

A communality cut-off value of 0.30 was applied, and 32 items were evaluated as sufficient. Values viewed in the factor correlation matrix were often <.15. First, principal component analysis, varimax rotation with an eigenvalue limitation (>1.0), maximum likelihood method, and scree plot were used. Principal axis factoring and promax rotation programs were run; however, promax rotation did not fit in this study. Finally, principal component analysis, varimax rotation, and scree plot were used to generate adequate factor numbers. As suggested by Osborne et al. (2008), communalities above 0.4 are acceptable when performing the EFA using principal axis factoring with promax rotation [26]. The number of factors was determined using a scree plot and the minimum average partial. The first 32-item EFA showed nine factors, with the cumulative explanation being 63.5%.

### Item reduction by combined EFA and CFA

To obtain the most adequate explanatory power using factor numbers, the EFA was run several times and received a powerful explanation in the fifth EFA. When the program was run using a factor number of 167 women, the communality of seven items (Q5, Q9, Q12, Q16, Q17, Q18, and Q32) among all 32 items (<.40 eigenvalue) was removed (Q5: “Bloody discharge from the vagina”; Q9: “My belly was sorely sick”; Q12: “My groin seemed to fall out”; Q16: “Lying down was stuffy, so I wandered around a bit”; Q17: “It was difficult to stand on the bus or train when commuting”; Q18: “Because a friend or relative came to the house, it made it difficult to clean or prepare food”; and Q32: “I was stressed because I could not walk”).

Subsequently, the EFA was conducted again with varimax rotation using the 25 remaining items, and Q4 (“Something like a runny nose came out of the vagina”; <.40 eigenvalue) was excluded. Twenty-four items and seven factors explained 63.4% of the total variance. The EFA was conducted several more times; six, four, and three factors explained 60.3% (KMO = .797, χ^2^ = 1363.77, *p* < .001), 49.6%, and 42.9% of the variance, respectively. Through the EFA evaluation, seven factors satisfied the KMO (>.80) and cumulative explanation of variance (>60%). Varimax rotation was conducted again using 24 items; Q19 (“When the uterine contractions disappeared, I went home”) only valued at .41 (<.50 eigenvalue) and was excluded. Finally, seven factors and 23 items explained 65.9% of the variance, and 167 participants satisfied the sampling adequacy for factor analysis (KMO) and Bartlett’s test of sphericity (KMO = .805, χ^2^ = 1322.52, DF 253, *p* < .001) (Table 2).

### Model fit

In this study, the fit of seven factors and 23 items explained the risk for PTB (root mean square residual [RMR] = .056, RMSEA = .043, NFI = .831, RFI = .746, IFI = .954, TLI = .925, CFI = .950; Table 3).

**Table 3:** Model fit of 23 items

Model	RMR	RMSEA	NFI	RFI	IFI	TLI	CFI
Default model	.056	.043	.831	.746	.954	.925	.950

### Reliability for internal consistency

Reliability was tested to select the 23 final items. Cronbach’s alpha for internal consistency was .85, with the high value indicating that it was the first scale to be developed. The 23 items had a mean of 34.08 ± 10.33 (*N* = 167, *F* = 61.71, *p* < .001). The mean CFA score of the PBRAS-23-K (*N* = 131) was slightly higher (36.88 ± 10.80) than that of EFA (*N* = 167). The PBRAS-23-K showed high internal consistency and satisfactory reliability (Cronbach’s alpha = .85), and the total mean ± SD for the PBRAS-23-K was 35.58 ± 10.35 in 298 participants. Except for five items [Q1 (.26), Q3 (.23), Q6 (.17), Q23 (.28), and Q30 (.11)], the internal consistency of the PBRAS-23-K had a corrected item-total correlation of over .30. Nonetheless, even if these five items were deleted, Cronbach’s alpha did not increase above .85, and reliability of the subscales was .60–.83, except for factor 6 (.44) and factor 7 (.20). Therefore, all 23 items were retained in this study (Table 4). The final version of the PBRAS-K comprised 23 items, as shown in Table S1. Table S2 presents the internal consistency of each item, whereas Figure S1 shows the seven dimensions of the PBRAS-23-K. Each item in the PBRAS-23-K was scored from 0 to 3, considering that a typical respondent was at or near the center of the Likert scale according to DeVellis [15]. The total score was used to determine the PTB risk level in women.

**Table 4:** Internal consistency of the PBRAS-23-K (*N* = 298)

PBRAS-K items		Corrected item-total correlation		Cronbach’s alpha if item deleted	
		***n* = 167**	***n* = 298**	***n* = 167**	***n* = 298**
Q1	1. I have anemia (hemoglobin level lower than 10 g/dL)	.26	.24	.85	.85
Q2	2. I feel depressed	.34	.47	.84	.84
Q3	3. I don’t take the prescribed medication	.23	.20	.85	.85
Q6	4. I cannot sleep well	.17	.23	.85	.85
Q7	5. My belly feels tight and hard often	.31	.31	.84	.84
Q8	6. I feel pelvic pressure	.53	.51	.84	.84
Q10	7. I feel deep penetrating pain	.49	.48	.84	.84
Q11	8. I have dull pain in my back and belly	.37	.41	.84	.84
Q13	9. I have lots of stress (at home/work)	.57	,59	.83	.83
Q14	10. I feel very sensitive (at home/work)	.62	.59	.83	.83
Q15	11. It is hard to work on my feet (at home/work)	.47	.48	.84	.84
Q20	12. I have too heavy of a workload (at home/work)	.50	.49	.84	.84
Q21	13. I have intense muscle pain	.55	.56	.84	.84
Q22	14. I’m worried about my baby being born too early	.44	.40	.84	.84
Q23	15. I try to hang tight even for one more day for my baby	.28	.27	.84	.85
Q24	16. I feel nervous to hear that I have a short cervix	.34	.29	.84	.85
Q25	17. I feel sad to hear that I could have preterm labor	.39	.42	.84	.84
Q26	18. I get stressed by hearing negative things from my doctor	.44	.38	.84	.84
Q27	19. I feel stressed by being responsible for all of the housework	.43	.47	.84	.84
Q28	20. I rest fewer than two hours a day	.33	.34	.84	.84
Q29	21. I get annoyed at my husband from time to time	.49	.52	.84	.84
Q30	22. I eat fewer than four times a day	.11	.18	.85	.85
Q31	23. What I want from my husband is not to do anything but to just listen to me, but I am sad he doesn’t understand it	.60	.55	.83	.84

*N* = 167: Cronbach’s alpha = .85 (mean ± SD = 33.98 ± 10.43) (total item mean = 1.48)

*N* = 131: Cronbach’s alpha = .85 (mean ± SD = 36.88 ± 10.80)

*N* = 298: Cronbach’s alpha = .85 (mean ± SD = 35.58 ± 10.35) (total item mean = 1.55)

Cronbach’s alpha = .84 (mean ± SD = 30.58 ± 9.21) (item mean = 1.53) for 20 items with Q3, Q6, and Q30 excluded

#### Convergent and criterion validity for construct validity

Validity was compared between the SPL-SAS and pregnancy-related stress. In terms of convergent validity, the PBRAS-23-K showed a significantly moderate correlation with pregnancy-related stress (*r* = .57, *p* < .001), indicating the high validity of a similar construct. With respect to concurrent validity, the PBRAS-23-K exhibited a low-to-moderate positive correlation [24] with RPD (*r* = .45, *p* < .001). As for criterion validity, SPL-SAS was evaluated for somatic symptoms, and PBRAS-23-K had a high correlation coefficient (*r* = .65, *p* < .001) (Table 5).

**Table 5:** Convergent and criterion validity *(N* = *298)*

Scales	SPL-SAS	HPRS	RPD	PBRAS-23-K
	***r* (*p*)**	***r* (*p*)**	***r* (*p*)**	
SPL-SAS	1			
HPRS	.47 (< .001)	1		
RPD	.53 (< .001)	-.29 (.001)	1	
PBRAS-23-K	.65 (< .001)	.57 (< .001)	.45 (< .001)	1

*SPL-SAS* Somatic Awareness Scale with Spontaneous Preterm Labor, *RPD* risk scoring for preterm delivery, *HPRS* High-risk pregnancy-related stress, *PBRAS* Preterm Birth Risk Assessment Scale

## Discussion

Measurement is a fundamental activity in science [27], with measurement scales being useful in evaluating attributes that cannot be directly measured. In the case of psychosocial attributes of stress or depression, the magnitude cannot be directly measured; however, pain can be calculated to some extent. In tool development research, it is necessary to plan the research by first deciding whether to perform only the EFA or CFA. Given that the EFA is data-driven and involves several subjective decisions, the CFA is therefore a more appropriate method for cross-validating the factor structure of a test. The basic question answered by the CFA is whether the factor structure matches the results of the original study [28].

To our knowledge, the structure of PTB-related variables and their relationships have not yet been elucidated; nevertheless, a few studies have investigated the predisposing factors for PTB. Maloni reported behavioral, environmental, demographic, medical, and reproductive factors [29]. Creasy et al. classified the socioeconomic status, previous medical history, daily habits, and aspects of the current pregnancy in RPD [6] but did not explain the concepts or theories behind PTB or PTL. Klockars-McMullen developed a scale based on the symptom perception model constructed by van Wijk and Kolk in 1997, who integrated the concepts of environment, experience, emotions, and other psychological variables into their symptom perception model [23]. Moreover, to date, few studies have utilized an integrative biopsychosocial model and only recommend its use in future studies [9, 30]. Social support, educational attainment, gestational diabetes, preeclampsia, barriers to healthcare, and psychopathology represent unidirectional pathways to PTL [31]. In 2016, Hoyman tested an integrative biopsychosocial model for PTL in Hispanic mothers of twins and those with preeclampsia and reported the number of prenatal care visits, prenatal emotional problems, and primipara-predicted PTL [32]. However, this model was biased for biomedical variables, such as preeclampsia, number of prenatal care visits, and primipara. Therefore, this model is not suitable for primigravida or pregnancies without pre-eclampsia. As the above findings did not establish a theory about PTB or PTL, this study thus focused on the combination of EFA and CFA. An EFA or CFA using the same sample is possible [28].

Other factors associated with PTB should also be considered. In 2020, Kim visualized nine components explaining PTB [8]. In this study, the first 32-item EFA revealed nine factors, with a cumulative explanation of 64.3% [KMO = .81 (>.80), χ^2^ = 1841.38, *p* < .001]. However, five repeated EFAs with a reduced number of items, as well as a scree plot, clearly showed seven factors and 23 items. In addition, from the perspective of item-total item correlation, the correlation coefficient of the item-total correlation was lower than .15, implying that this scale might not be a complete version. Therefore, testing other sample groups is necessary. In this study, the fit of the seven factors and 23 items of the PBRAS-K explained the risk for PTB (RMR = .56, RMSEA = .043, TLI = .925, CFI = .950); These values satisfied cutoff criteria for fit indices [20], that is, the PBRAS-23-K was valid and reliable at this evaluation stage.

Pregnant women with PTL are at risk for giving birth prematurely; consequently, they are hospitalized and managed in MFICUs. In MFICUs, patients are required to rest, and an anti-contraction drug is administered to prevent labor progression. The patients are discharged when uterine contractions disappear. While admitted, women with PTL are not concerned about the hard work that they perform at home (or workplace) or how stressed they were. When they go home, they are again in a similar stressful situation, and many return to the hospital within a few days.

### Reliability and validity of measurement

In this study, the internal consistency showed a Cronbach’s alpha of .85 for the total items, indicating an adequate instrument [33]. Furthermore, the PBRAS-23-K retained items with *r* < 0.3 (corrected item-total correlation) for broad measurements. As suggested by Clark and Watson in 1995, the item-total correlation items (0.15–0.20) should be selected for broad measurements, and items with >0.40 are recommended for narrow measurements [34]. In the subscales, factors 1, 2, 3, and 4 were reliable, whereas the reliability of factors 6 and 7 was .44 and .20, respectively. These two factors and four items did not affect internal consistency. While factors comprising small items might have low reliability [28], the PBRAS-23-K should be studied with larger sample sizes. The PBRAS-23-K showed high validity with a construct similar to that of the high-risk pregnancy-related stress assessment (*r* = .57, *p* < .001) and exhibited a low-to-moderate positive correlation [24] with RPD (*r* = .45, *p* < .001). Rea and Parker reported that the *r*-value (0.4–0.6) indicated a moderately strong positive relationship [33]; however, evaluations might slightly differ between researchers. The PBRAS-K also showed a high correlation with the SPL-SAS for somatic symptoms (correlation coefficient *r* = .65, *p* < .001). Except for Q28 (*r* = .48), a particular construct correlated with other tests that assessed the same construct (≥.50) but did not correlate with tests that measured different constructs (<.33). In other words, the PBRAS-23-K had high convergent and concurrent validity.

### Recommendations for future studies

The PBRAS-23-K exhibits a partial correspondence with the seven factors elucidated using the generalized methodology with machine learning modelling as reported by Della Rosa et al. [35]. The rationale behind the omisson of six out of the seven factors in the PBRAS-23-K pertains to a deliberate decision made during the developmental phase of the scale construction. The exclusion of these six factores resulted from the methodology’s focus on streamlined “yes/no” responses, as they primarily encompassed health conditions or medical issues. Consequently, the the PBRAS-23-K predominantly comprises items with multiple responses of degree. In light of this, it is recommended that future research considers a comprehensive integration of the PBRAS-23-K alongside the seven factors delineated by Della Rosa et al. [35]. This collaborative approach has the potential to yield a more robust and multifaceted framework for assessing PTB risk. Also, in this study the degree of PTB risk was not analyzed. The PBRAS-K cut-off scores for minimal, mild, moderate, and severe PTB risk should be established with statistical models using machine learningin future studies.

### Study limitations

In this study, the researcher adopted a minimum sample size of five times the number of items for the CFA based on a previous study [28]. In this study, sample sizes of 167 for the 32-item EFA and 132 for the 23-item CFA were deemed to be suitable. The item-total item correlation was over 0.3 in this study. Therefore, the ratio of the sample size to the total items was approximately 5, and it was reasonable to use this ratio. The recommended sample size has also been inconsistent among researchers, and larger samples are required for more stable scales [36]. For a more rigid CFA evaluation, the use of larger sample sizes than the general rule may be necessary, and repeating and exploring the optimal reductions in the number of observed variables may also be required.

### Implications

The PBRAS-23-K was developed using seven factors based on a previous study, which reported that PTB had nine components. This scale should be further simplified for clinical and public nurses to counsel, educate, and provide care to women at risk for PTB. The author is currently developing a PTB risk assessment App using the items from PBRAS-K.

## Conclusion

The PBRAS-23-K is a valid and reliable instrument for assessing the risk for PTB in pregnant women. Clinical nurses and obstetricians should be encouraged to obtain information regarding effective interventions in MFICUs. This scale provides meaningful results and reflects the opinions of women who had experienced PTB. In the future, the scale should be evaluated for standardization and cut-off scores using larger sample sizes and is suggested to be used for the components of PTB modeling.

### Supplementary Information

**Additional file 1:** **Table S1.** Preterm Birth Risk Assessment Scale-Korean version (PBRAS-23-K).

**Additional file 2:** **Table S2.** Internal consistency of each factor.

**Additional file 3:** **Figure S1.** Confirmatory factor analysis.

## Data Availability

All data generated or analyzed in this study are included in the article and are available from the author Kim J. upon reasonable request.

## Abbreviations

CFA: Confirmatory factor analysis
CFI: Comparative fit index
EFA: Exploratory factor analysis
IFI: Incremental fit index
KMO: Kaiser–Meyer–Olkin test
MFICU: Maternal–fetal intensive care unit
NFI: Normed fit index
PTB: Preterm birth
PTL: Preterm labor
RFI: Relative fit index
RMSEA: Root mean square error of approximation
SPL-SAS: Somatic Awareness Scale with Spontaneous Preterm Labor
TLI: Tucker-Lewis index
