# Supplementary material for: Development and validation of the 23-item preterm birth risk assessment scale-Korean version
Source: BMC Pregnancy Childbirth. 2023 Sep 16;23:668. doi: 10.1186/s12884-023-05975-x (PMC10504700; doi:10.1186/s12884-023-05975-x)
Supplement: Supplementary file 1 — Additional file 1: Table S1. Preterm Birth Risk Assessment Scale-Korean version (PBRAS-23-K). [file 12884_2023_5975_MOESM1_ESM.docx]

**Table S1.** **The Preterm Birth Risk Assessment Scale-Korean Version [PBRAS-23-K]**

We would like to evaluate the preterm labor risk by assessing what symptom(s) you experienced ***during your pregnancy,*** specifically from the 20^th^ week of pregnancy to labor. Please check **V** for the following questions that apply to you.

| \| **Questions** \| **Never**  **(0)** \| **Rarely**  **(1)** \| **Sometimes**  **(2)** \| **Often**  **(3)** \|  \| \| --- \| --- \| --- \| --- \| --- \| --- \| \| 1. I have anemia (hemoglobin level lower than 10 g/dL). \|  \|  \|  \|  \|  \| \| 2. I feel depressed. \|  \|  \|  \|  \|  \| \| 3. I don’t take the prescribed medication. \|  \|  \|  \|  \|  \| \| 4. I cannot sleep well. \|  \|  \|  \|  \|  \| \| 5. My belly feels tight and hard often. \|  \|  \|  \|  \|  \| \| 6. I feel pelvic pressure. \|  \|  \|  \|  \|  \| \| 7. I feel deep penetrating pain. \|  \|  \|  \|  \|  \| \| 8. I have dull pain in my back and belly. \|  \|  \|  \|  \|  \| \| 9. I have lots of stress (at home/work). \|  \|  \|  \|  \|  \| \| 10. I feel very sensitive (at home/work). \|  \|  \|  \|  \|  \| \| 11. It is hard to work on my feet (at home/work). \|  \|  \|  \|  \|  \| \| 12. I have too heavy of a workload (at home/work). \|  \|  \|  \|  \|  \| \| 13. I have intense muscle pain. \|  \|  \|  \|  \|  \| \| 14. I’m worried about the baby being born too early. \|  \|  \|  \|  \|  \| \| 15. I try to hang tight even for one more day for my baby. \|  \|  \|  \|  \|  \| \| 16. I feel nervous to hear that I have a short cervix. \|  \|  \|  \|  \|  \| \| 17. I feel sad to hear that I could have preterm labor. \|  \|  \|  \|  \|  \| \| 18. I get stressed by hearing negative things from my doctor. \|  \|  \|  \|  \|  \| \| 19. I feel stressed by being responsible for all of the housework. \|  \|  \|  \|  \|  \| \| 20. I rest fewer than two hours a day. \|  \|  \|  \|  \|  \| \| 21. I get annoyed at my husband from time to time. \|  \|  \|  \|  \|  \| \| 22. I eat fewer than four times a day. \|  \|  \|  \|  \|  \| \| 23. I am sad that my spouse doesn’t understand what I feel difficulty. \|  \|  \|  \|  \|  \| |
| --- | --- | --- | --- | --- | --- | --- | --- | --- | --- | --- | --- | --- | --- | --- | --- | --- | --- | --- | --- | --- | --- | --- | --- | --- | --- | --- | --- | --- | --- | --- | --- | --- | --- | --- | --- | --- | --- | --- | --- | --- | --- | --- | --- | --- | --- | --- | --- | --- | --- | --- | --- | --- | --- | --- | --- | --- | --- | --- | --- | --- | --- | --- | --- | --- | --- | --- | --- | --- | --- | --- | --- | --- | --- | --- | --- | --- | --- | --- | --- | --- | --- | --- | --- | --- | --- | --- | --- | --- | --- | --- | --- | --- | --- | --- | --- | --- | --- | --- | --- | --- | --- | --- | --- | --- | --- | --- | --- | --- | --- | --- | --- | --- | --- | --- | --- | --- | --- | --- | --- | --- | --- | --- | --- | --- | --- | --- | --- | --- | --- | --- | --- | --- | --- | --- | --- | --- | --- | --- | --- | --- | --- | --- | --- | --- |
