# Supplementary material for: Development and validation of the 23-item preterm birth risk assessment scale-Korean version
Source: BMC Pregnancy Childbirth. 2023 Sep 16;23:668. doi: 10.1186/s12884-023-05975-x (PMC10504700; doi:10.1186/s12884-023-05975-x)
Supplement: Supplementary file 2 — Additional file 2: Table S2. Internal consistency of each factor. [file 12884_2023_5975_MOESM2_ESM.docx]

**Table S2. Internal Consistency of Each Factor**

|  | | | | |
| --- | --- | --- | --- | --- |
| Factor | No. of item (item number) | Mean ± SD | Item’s Mean ± SD | Cronbach’s α |
| A1 | 5 (Q22, Q23, Q24, Q25, Q26) | 9.34±3.86 | 1.87 ±1.09 | .80 |
| A2 | 4 (Q13, Q14, Q15, Q20) | 9.04±3.01 | 1.57± 0.85 | .83 |
| A3 | 3 (Q27, Q29, Q31) | 3.83±2.46 | 1.28±0.97 | .78 |
| A4 | 4 (Q7, Q8, Q10, Q11) | 5.70±2.65 | 1.43±0.81 | .72 |
| A5 | 3 (Q1, Q3, Q21) | 1.91±1.92 | 0.64±0.73 | .61 |
| A6 | 2 (Q2, Q6) | 3.56±1.34 | 1.78±0.70 | .44 |
| A7 | 2 (Q28, Q30) | 3.38±1.52 | 1.69±1.03 | .20 |
| Total | 23 | 33.98±10.43 | 1.48±0.90 | .85 |
| SD: standard deviation | | | | |
