# Supplementary figures and images for: Development and validation of the 23-item preterm birth risk assessment scale-Korean version
Source: BMC Pregnancy Childbirth. 2023 Sep 16;23:668. doi: 10.1186/s12884-023-05975-x (PMC10504700; doi:10.1186/s12884-023-05975-x)

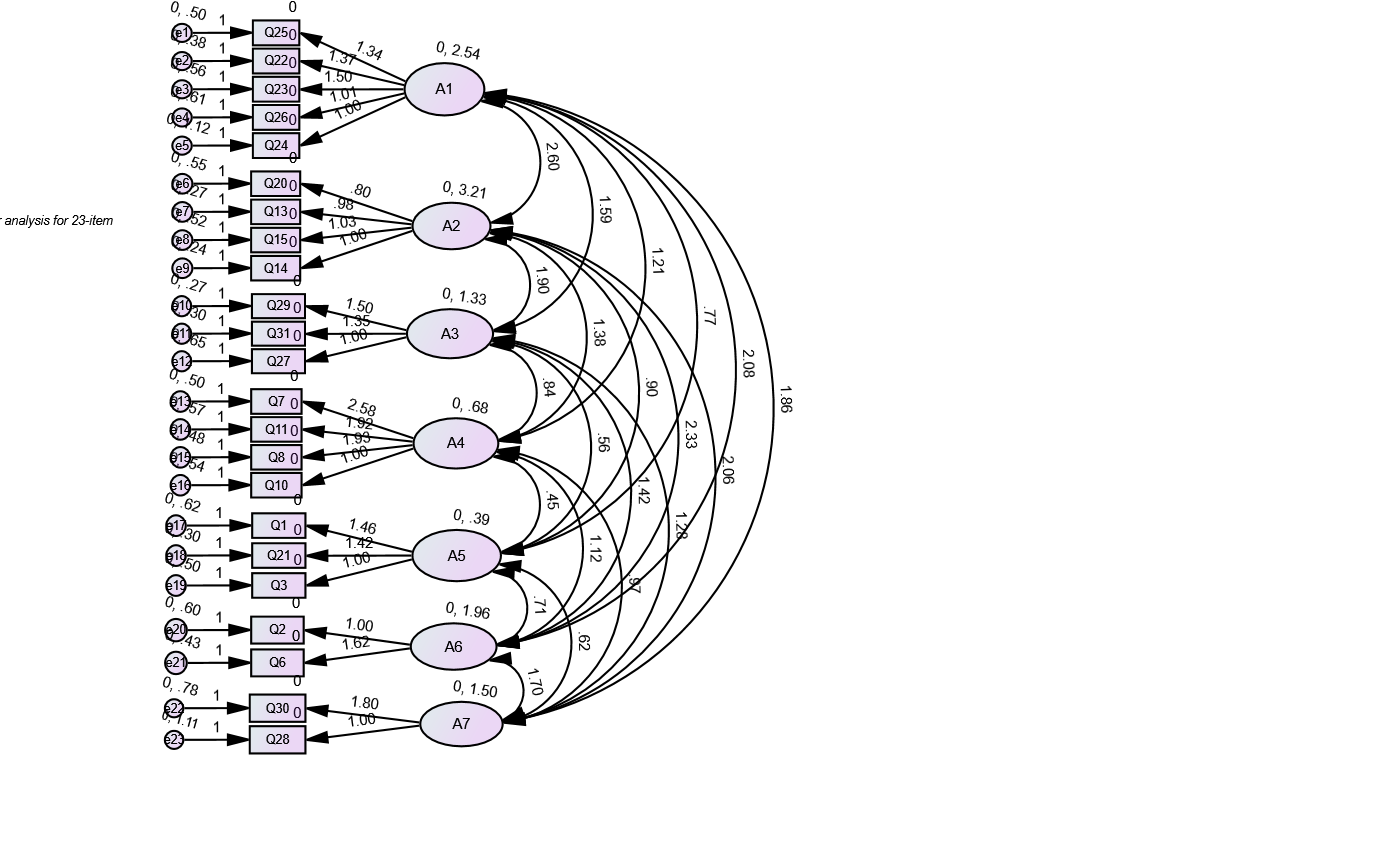


Figure S1. Confirmatory factor analysis

Supplement: Supplementary file 3 — Additional file 3: Figure S1. Confirmatory factor analysis. [file 12884_2023_5975_MOESM3_ESM.docx]
